# Perspectives of Premedical Students at a Medical University College in Malaysia Regarding Augmented Reality Integration in Anatomy Education: Cross-Sectional Study

**DOI:** 10.2196/64402

**Published:** 2026-06-08

**Authors:** Fadhilah Idris, Nur Hidayah Mohd Noor, Faidruz Azura Jam, Nurul Huda Mohd Nor, Siti Fadziyah Mohamad Asri

**Affiliations:** 1 Centre for Foundation and General Studies, Manipal University College Malaysia, Persimpangan Batu Hampar, Bukit Baru, Malacca, 75150, Malaysia, +60 193005759; 2 Department of Biochemistry, Faculty of Medicine, Manipal University College Malaysia, Malacca, Malaysia; 3 Department of Human Anatomy, Faculty of Medicine and Health Sciences, Universiti Putra Malaysia, Selangor, Malaysia; 4 Department of Anatomy and Physiology, Faculty of Medicine, Universiti Sultan Zainal Abidin, Terengganu, Malaysia

**Keywords:** premedical students, augmented reality, AR, interactive learning, anatomy education

## Abstract

**Background:**

For novice anatomy learners, studying human anatomy using textbooks, 2D learning materials, and static anatomical models frequently causes challenges in understanding complex anatomical structures. Since access to dissected human donor bodies is limited in many premedical programs, researchers are concerned with exploring novel supplementary approaches to anatomy learning. This research explores the effectiveness of an augmented reality (AR) app in enhancing the anatomy learning experiences of premedical students.

**Objective:**

This cross-sectional study was conducted to evaluate premedical students’ perceptions of AR integration in early anatomy learning and to examine whether these perceptions differ according to the students’ demographic variables.

**Methods:**

An AR app was introduced to premedical students during anatomy practical sessions. Participants were divided into groups of 4, and a volunteer from each group wore a garment that displayed dynamic anatomical visuals when scanned with a smartphone or tablet. Participants then evaluated their anatomy learning experiences using a survey questionnaire after using the AR app for 1 hour.

**Results:**

A total of 284 participants were included in the study. Based on the participants’ ratings of the anatomy learning app with AR, the overall perception score was high (median 4.92, IQR 4.17‐5.00). A statistically significant difference in perception score was observed between male and female students (ρ=0.212; *P*<.001), with female students reporting more favorable opinions of the AR app. However, no statistically significant associations were observed between perception and age (ρ=0.034; *P*=.57), student batch number (ρ=-0.009; *P*=.88), or monthly household income (ρ=−0.041; *P*=.49).

**Conclusions:**

By providing interactive learning experiences, AR is positively perceived among diverse premedical student backgrounds; thus, it has broad potential to serve as a valuable supplementary tool to complement conventional teaching practices for novice anatomy learners.

## Introduction

Human anatomy education is an essential component of medical training, providing the foundational knowledge necessary for clinical practice and diagnosis. However, traditional methods of teaching anatomy, primarily reliant on 2D textbook images, photographic projections of lecture slides, static anatomical models, and flashcards, often fail to effectively convey the complex 3D relationships between structures [1-3]. While human donor body dissection remains the gold standard for anatomy training in medical schools [4,5], donor body dissection has several drawbacks, such as restricted access [6-8], time constraints [9], financial limitations [9,10], and ethical concerns [10-12]. These challenges are particularly prominent in the context of foundation-level students in Malaysia, where public medical schools face constraints in providing adequate dissection facilities and incorporating this method into early anatomy courses [6]. Although private institutions may have more resources, sourcing human donor bodies and integrating dissection into premedical curricula remains difficult in many cases.

In the last few years, many educational institutions have progressively explored technology-enhanced learning approaches to supplement traditional anatomy instruction. Following the COVID-19 pandemic, digital learning tools have gained prominence with the accelerated adoption of technology in higher educational institutions [13,14]. Even though online platforms such as learning management systems and video-conferencing tools like Zoom (Zoom Communications, Inc), Microsoft Teams, and Google Meet enabled the continuation of teaching and learning sessions during the COVID-19 pandemic [15,16], as well as postpandemic periods, these platforms mostly supported content delivery rather than interactive visualization of anatomical structures. Consequently, educators have sought advanced digital tools that can improve spatial learning and enhance student engagement in anatomical education.

Among the myriad of emerging technologies, augmented reality (AR) has stood out as a technology that enhances students’ engagement and understanding of complex scientific concepts. AR incorporates computer-generated content into real-world elements, permitting users to experience immersive learning environments that overcome the limitations of traditional approaches [17]. In the context of anatomy education, AR allows students to visualize 3D representations of various human body systems and internal organs without the requirement for physical specimens. This capability enables students to explore anatomical structures dynamically through multiple functions such as zooming, rotation, and layered visualization, potentially enhancing spatial understanding and conceptual comprehension [18].

Another significant benefit of AR technology over other extended reality technologies, such as virtual reality and mixed reality, is its accessibility through personal devices such as smartphones and tablets [19]. This permits AR-based learning approaches to be incorporated not only into classroom-based but also into self-directed learning practices [20], without requiring expensive, specialized, or tethered headsets, which are crucial for virtual reality and mixed reality technologies. Previous studies have reported that AR-based learning apps can improve student engagement, interest, and understanding of complex scientific concepts [19,21,22]. Consequently, AR is progressively considered an effective supplementary tool that can support traditional anatomy teaching methods.

However, the effectiveness of AR in enhancing anatomy education, especially in the context of premedical students, remains underexplored. A conceptual framework grounded in educational theories for AR-powered teaching is essential to assess its effectiveness in improving student learning outcomes. This study is informed by the perspectives of Kirkwood and Price’s [23] review, which conceptualizes enhancement in relation to the nature of technology intervention, how improvement in learning is defined (operational, quantitative, or qualitative), and the level of evidence used to support such claims. Within this framework, AR is considered to enhance learning primarily through qualitative improvements in the learner experience, including improved spatial visualization, interactivity, and engagement with anatomical content. Accordingly, this study focuses on the learner experience dimension and operationalizes enhancement through students’ perceived educational value of AR, treated as a unified construct reflecting usability, engagement, and perceived learning support. The use of self-reported perception is consistent with prior technology-enhanced learning research as an appropriate early-stage evaluative approach, particularly for interventions that supplement existing teaching practices. However, this represents a lower level of evidence and is subject to inherent limitations, including potential response bias and the absence of direct measurement of learning outcomes.

Although AR is increasingly used in medical education, there is still limited understanding of how premedical students experience and perceive its integration into early anatomy learning, particularly in resource-constrained settings where traditional hands-on approaches are limited. Hence, this study aimed to evaluate premedical students’ perceptions of AR integration in early anatomy learning, conceptualized as a single perception construct. Specifically, we asked whether students view AR as an acceptable and useful aid tool for learning anatomy and whether this perception varies across demographic variables. This was examined using a cross-sectional study with a structured and validated questionnaire. We hypothesized that students would generally report a positive perception of AR integration regardless of demographic background. The findings from this study may inform curriculum design and guide evidence-based decisions for educators, curriculum designers, and academic administrators regarding the integration of AR in anatomy education, particularly in settings with limited access to conventional anatomical teaching resources.

## Methods

### Ethical Considerations

Ethical approval was obtained from the Research Ethics Committee of Manipal University College Malaysia (MUCM; 035/2023) prior to the commencement of the research study. This study met the criteria given its educational nature with no potential risks to participants. To ensure the highest standards of ethical research in educational contexts, the study incorporated comprehensive measures in accordance with established guidelines. Informed consent was acquired from the participating students or their parents or legal guardians to ensure their understanding of the purpose of the study, its procedures, any side effects, and any benefits of being in this study. To further ensure the confidentiality and anonymity of the participants, no personal identifiers were collected in this study. All data were recorded anonymously, and access to the collected dataset was restricted to authorized members of the research team for data management and data analysis purposes. Participation in this study was voluntary, and participants had the right to refuse to answer any questions in the questionnaire. Participants also had the right to withdraw from this study at any time, and refusal to participate or withdrawal from this study did not affect any benefits to which they were otherwise entitled. By implementing these ethical considerations, the research study has not only protected the rights, safety, and well-being of the participants but also upheld the credibility and integrity of the research findings.

### Participants

This research study was conducted on premedical students attending a Basic Human Anatomy course in semester 2 of the Foundation in Science (FiS) program at MUCM. The study population comprised students enrolled in the FiS program at the medical university college during the 2023/2024 academic year, including FiS Batch 27, FiS Batch 28, and FiS Batch 29. The term “Batch” refers to the student cohort within the premedical program, typically categorized according to the intake period (ie, entry group). A total of 3 student cohorts or batches from the 2023/2024 academic year were included in this study, whereby FiS Batch 27, FiS Batch 28, and FiS Batch 29 enrolled in the premedical program in April 2023, July 2023, and August 2023, respectively. Upon completion of this 1-year FiS program, the students will pursue their undergraduate studies in MBBS in the upcoming academic year. Before the students’ enrollment in this FiS program, these students either completed the International General Certificate of Secondary Education or the Malaysian Higher School Certificate, and thus, they had basic science knowledge of biology, chemistry, and physics.

One of the compulsory courses in semester 2 of the FiS program is Basic Human Anatomy, which is designed to enhance the students’ basic knowledge of the human body. The topics covered in this course include an introduction to anatomy and the human body, the introduction of different types of human body tissues and their histological features, and various topics in systemic anatomy, such as the skeletal, nervous, respiratory, cardiovascular, digestive, and urinary systems. The methods of content delivery were mainly didactic lectures, tutorials, and practical sessions facilitated by qualified instructors.

During week 3 of the Basic Human Anatomy course, after the introduction to anatomy and the human body, as well as the introduction of different types of human body tissues and their histological features, topics had been delivered, the participants were introduced to the Virtuali-Tee AR app in focused sessions. These were formal practical sessions, and the participants were guided by qualified instructors to interact with the AR app in groups for 1 hour to promote collaborative learning.

All the FiS student batches received the same curriculum and teaching content and participated in the same AR-based practical session, delivered using a standardized protocol, ensuring consistency and comparability across student cohorts despite variations in their intake periods.

### Sample Size

This cross-sectional study was conducted among premedical students enrolled at MUCM during the 2023/2024 academic year, with a total eligible population of 293 students. The minimum required sample size was determined using the formula for estimating finite population proportion with an assumption of a 95% CI, a margin of error of 5%, and a maximum response distribution of 50%. This calculation yielded a required sample size of 167 respondents. The researchers anticipated a 30% nonresponse rate; therefore, the final adjusted sample size was set at 239 students. In this study, a total of 284 students voluntarily participated, exceeding the sample size requirement and thereby ensuring adequate statistical power for data analysis. The larger sample size also allows for more robust subgroup analyses across demographic variables, including age, gender, student batch number, and monthly household income.

### Study Design

**Figure 1.**
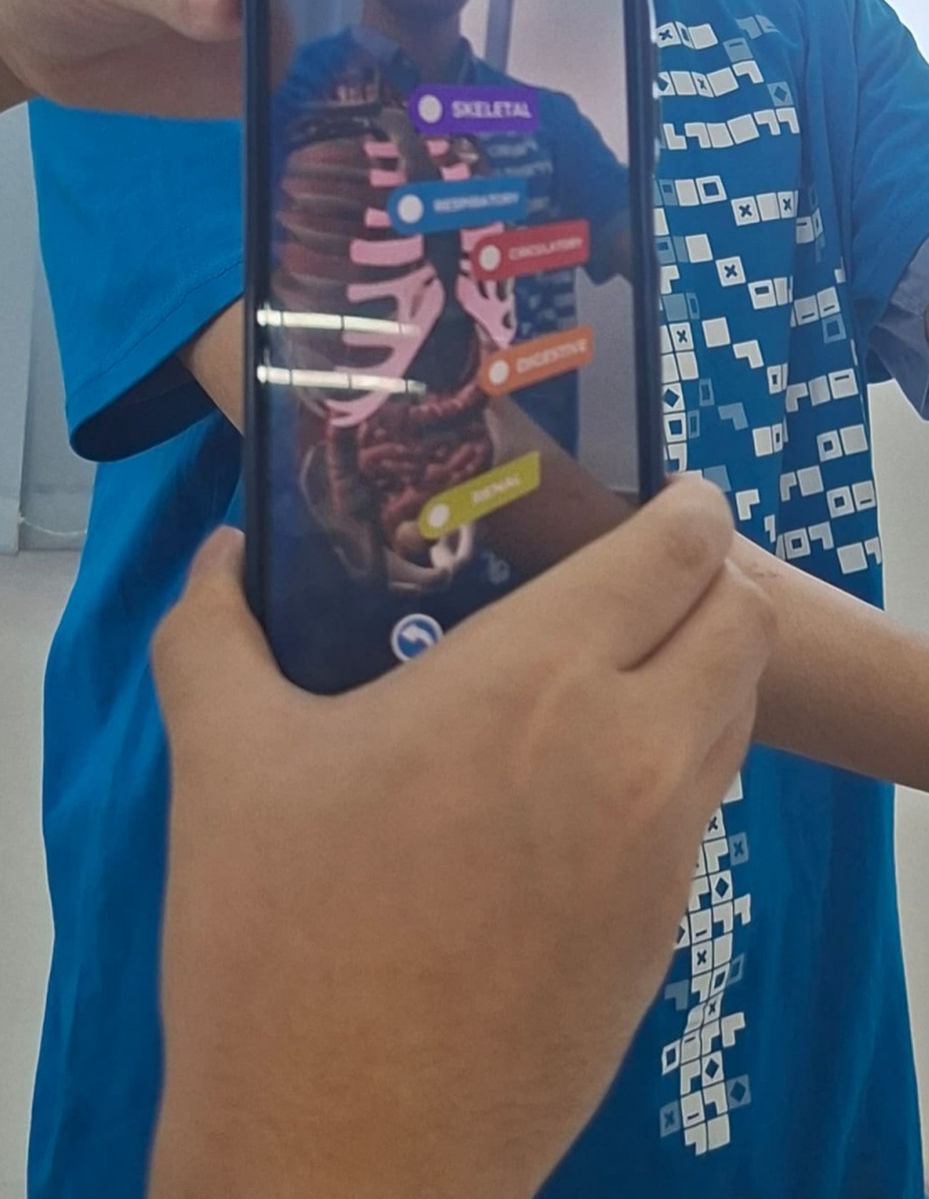
Upon scanning the garment with smartphones or tablets, the Virtuali-Tee augmented reality (AR) app reveals interactive anatomical graphics and visuals.

This study involved researchers, who are experienced and qualified instructors, in introducing premedical students to the Virtuali-Tee AR app, which was developed by Curiscope (Figure 1). This app has been purchased by MUCM and is available on the App Store and Google Play Store. The researchers instructed the participants to install the AR app on their smartphones or tablets during the AR-based practical sessions. These sessions were carried out with structured learning objectives, including: (1) identification of basic anatomical structures, (2) visualization of 3D anatomical relationships, and (3) familiarization with the AR app’s functional features (eg, rotation, zooming, and organ description). These standardized sessions commenced with a brief orientation given by the researchers, followed by a guided demonstration of the AR app’s functionality. The researchers then supervised the participants as they engaged in hands-on exploration of the AR app within a 1-hour time frame. During this time frame, a few volunteers among the participants wore a piece of garment that, when scanned with smartphones or tablets, superimposed anatomical graphics and visuals on their bodies. The researchers were present throughout the sessions to guide the participants and ensure alignment with the learning objectives of the sessions. For the Virtuali-Tee AR app, the garment integrated a unique pattern of a ribcage as a marker, enabling the app to track participants’ body positions and orientations accurately. This ensured that the overlaid 3D anatomical visuals remained precisely aligned with the participant’s body movements in real-time, while providing a unique and immersive educational experience.

**Figure 2.**
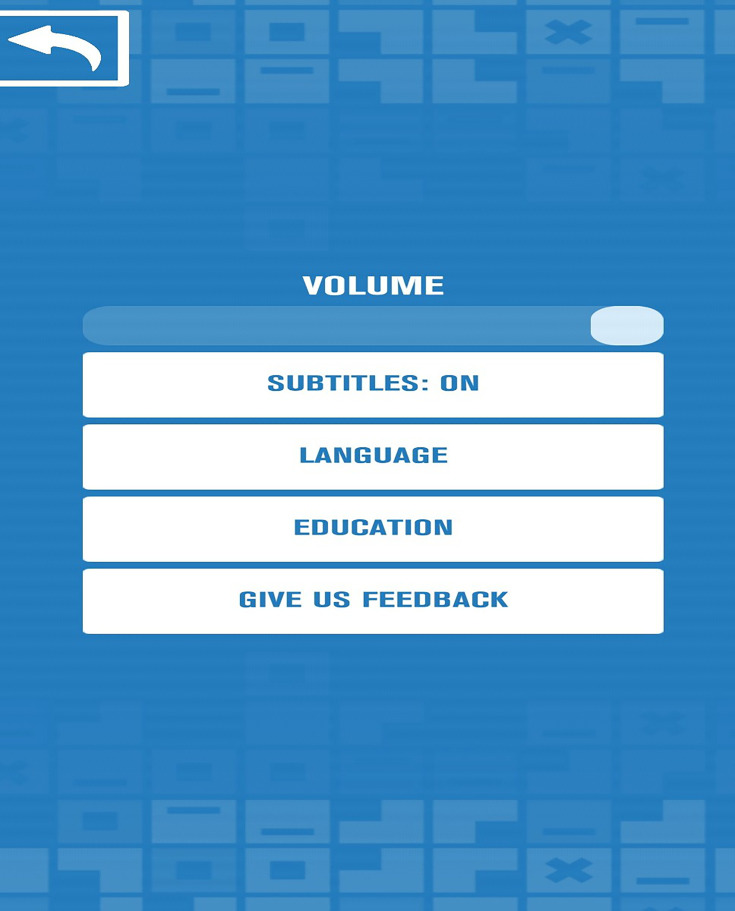
The settings section of the app enabled the participants to adjust the sound volume, activate subtitles, and choose their preferred language.

**Figure 3.**
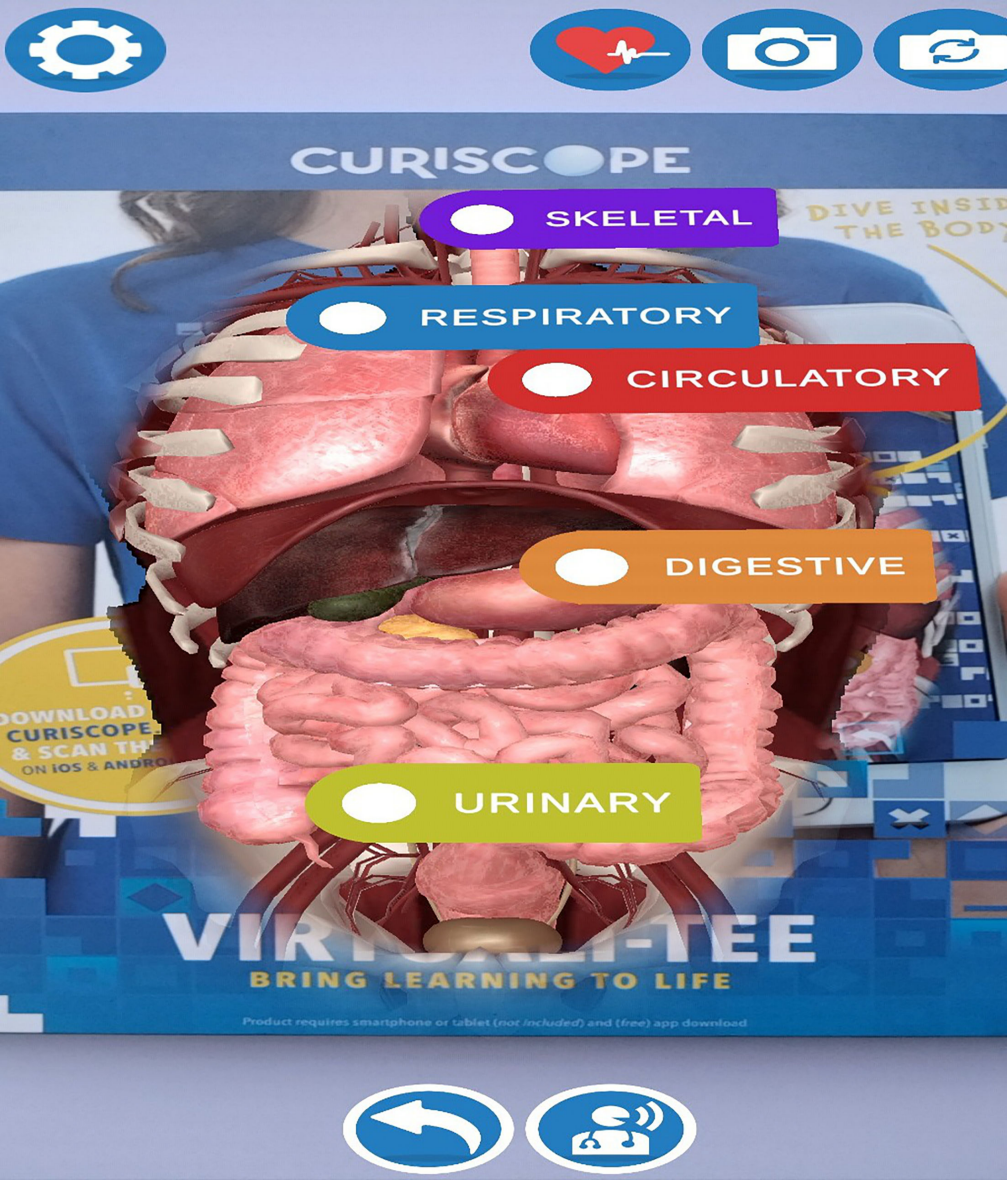
The 5 different human body systems are further detailed as participants clicked on the various interactive functions.

**Figure 4.**
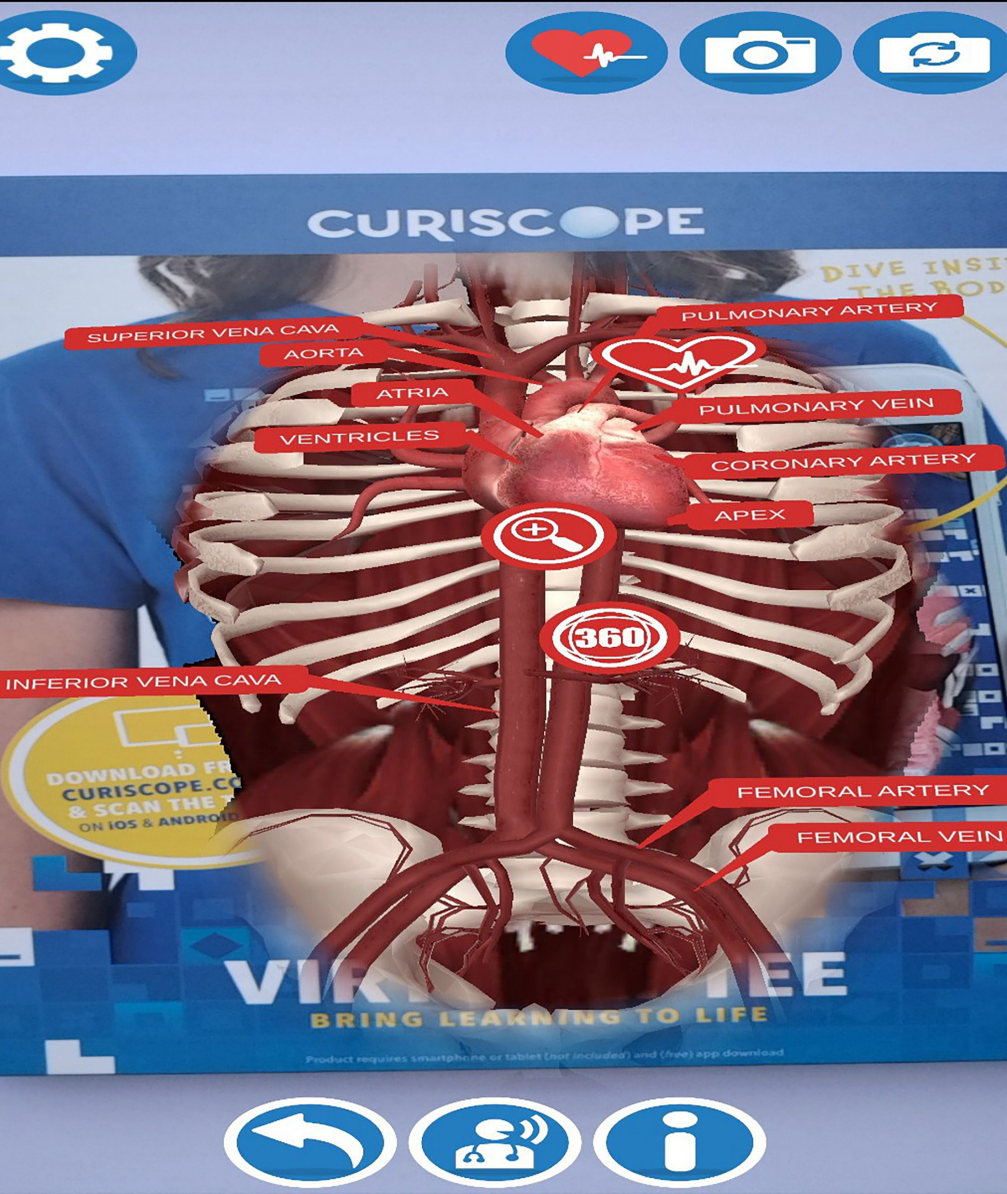
Various organs of the circulatory system were further explained as participants clicked on the 3D organ models featured in the app.

**Figure 5.**
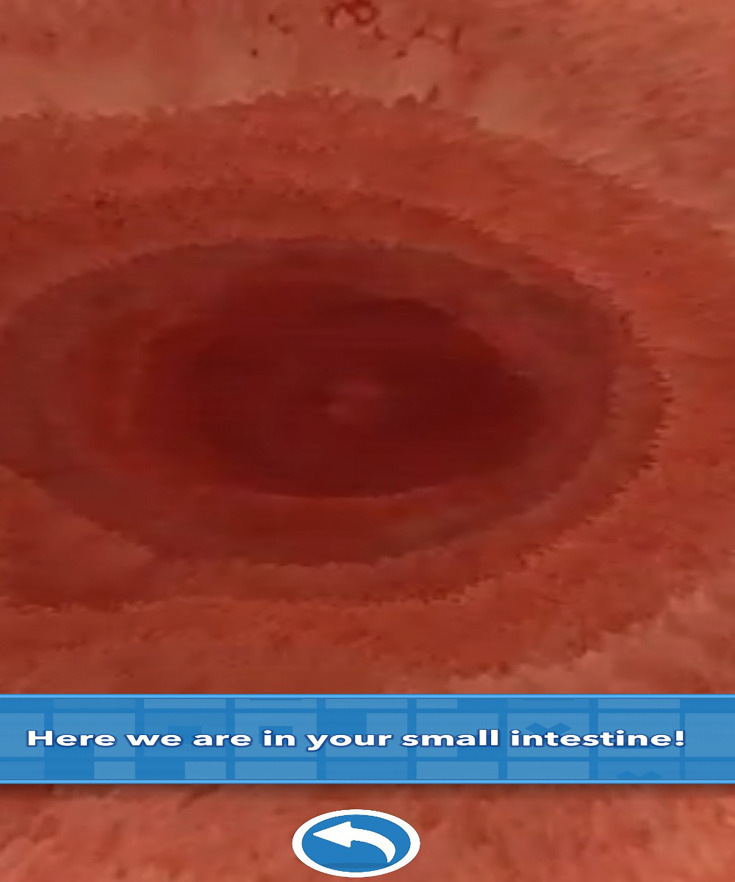
The 360-degree view function enabled the participants to visualize the inner wall of the small intestine at the microscopic level.

**Figure 6.**
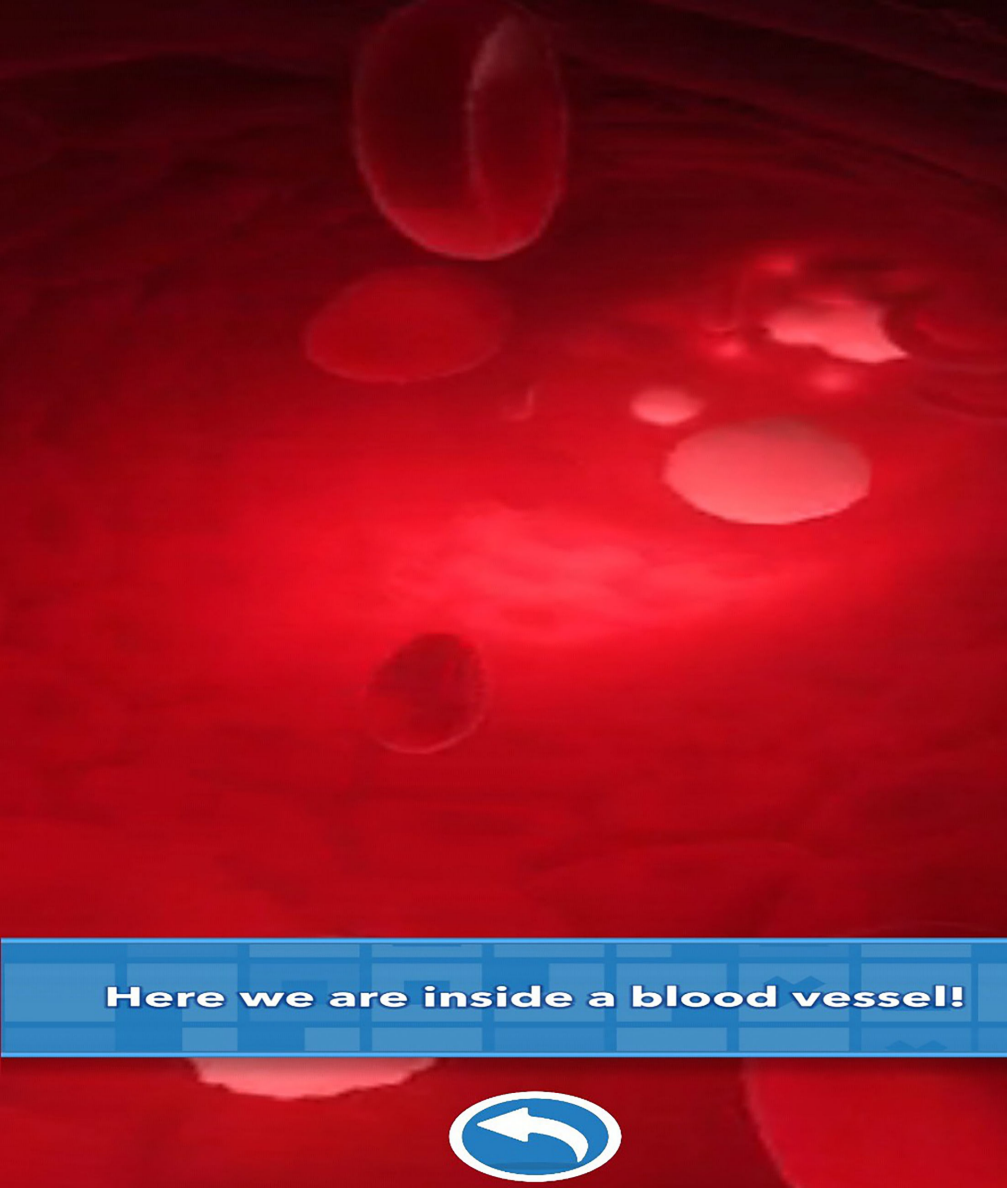
Participants had the opportunity to virtually dive into the blood circulation, enabling them to observe different blood components and formed elements.

In the Virtuali-Tee AR app settings, participants had the option to adjust sound volume, activate subtitles, and choose their preferred speech and text language (Figure 2). Participants were able to interact with various features within the app, allowing them to navigate and explore multiple systems of the human body, namely the skeletal, respiratory, circulatory, digestive, and urinary systems (Figure 3), by using their personal devices. This app enabled participants to locate the positions of internal organs (Figure 4) and perform gross observations of the organs from different angles. By clicking on the 3D organ models featured in the app, comprehensive information about the organs appeared. The 360° view function embedded in the app enabled participants to explore detailed structures and experience immersive virtual learning as they examined the internal wall of the small intestine (Figure 5), the circulation of blood (Figure 6), and the surface of alveoli at the microscopic level.

The Virtuali-Tee AR content was designed to provide precise depictions of internal anatomical structures by focusing primarily on the thoracic and abdominal regions while avoiding inappropriate details in other sensitive regions of the body. Additionally, this app required active participation from all participants, with a volunteer from each group choosing to wear the garment and providing informed consent. These measures aligned with ethical guidelines in educational technology, ensuring that the use of the Virtuali-Tee AR garment respected participants’ dignity and promoted a safe teaching and learning environment. Following the 1-hour AR-guided practical sessions, the researchers distributed an online survey questionnaire to the participants to evaluate their anatomy learning experiences with the AR app.

### Data Collection

The data were collected through an online survey questionnaire administered to premedical students who participated in the research study. The survey questionnaire, hosted on Google Forms, was shared with participants electronically. Responses were collected online and stored in a secure online database. No manual data entry was performed. Both questionnaire items and response options were randomized using the built-in shuffle functions in Google Forms. There were 2 sections in the questionnaire, which consisted of 5 pages. The first section gathered demographic information such as age, gender, student batch number, and monthly household income. In the second section, a questionnaire adapted from a previous study [1] was used. Data completeness was assessed both during and after survey submission. As a dynamic completeness check, each question required a response before the respondent could proceed to the next page. While responding to the questionnaire, the back button and response editing were enabled. The survey was open from September 6, 2023, to January 9, 2024. Participation was voluntary, and participants could exit the survey at any point before submission.

### Online Survey Administration

The online survey was reported in accordance with the CHERRIES (Checklist for Reporting Results of Internet E-Surveys) guidelines (Checklist 1) [24]. The questionnaire was administered using Google Forms as a closed survey and was accessible only to eligible premedical students through an electronic invitation sent by the researchers. Prior to accessing the questionnaire, participants were presented with an information page describing the study’s purpose, voluntary participation, confidentiality of responses, and estimated completion time. Proceeding with the survey implied informed consent. No incentives were offered for participation. To prevent multiple submissions, responses were restricted to a single entry per participant.

### Data Collection Tool

The questionnaire used in this study was adapted from a previously published survey instrument [1]. The questionnaire comprised 12 items rated on a 5-point Likert scale, ranging from 1 (“strongly disagree”) to 5 (“strongly agree”). The researchers measured the construct validity and internal consistency reliability of the adapted questionnaire to assess its psychometric properties within this study population.

The construct validity was evaluated using exploratory factor analysis. The exploratory factor analysis demonstrated that all items in the survey instrument had high communality values (>0.80), suggesting that a substantial proportion of the variance in each item was shared with the extracted factor. This important finding indicated that the items in the survey instrument predominantly measured a single latent construct rather than multiple distinct domains. Based on these important findings, the survey instrument was treated as a single construct in the subsequent data analyses.

On the other hand, the researchers evaluated the internal consistency reliability using Cronbach α. The overall Cronbach α value was 0.96, indicating high internal consistency and reliability, and further supporting the interpretation that the questionnaire items collectively measured a 1D construct.

Additional forms of validity testing, including predictive and discriminant validity, were not performed. The researchers decided this because the survey instrument was intended to capture students’ self-reported perceptions rather than to predict objective learning outcomes or differentiate between theoretically distinct constructs.

### Data Analysis

The researchers included only the fully completed questionnaires in the final analysis. Of the 293 eligible students, 284 completed the online questionnaire, yielding a completion rate of 96.9%. Data analysis was conducted using IBM SPSS Statistics version 29.0. For the first section of the questionnaire, descriptive statistics were performed on the demographic data of the study participants, and the data were reported as frequencies and percentages. In the second section, a Likert scale ranging from 1 (“strongly disagree”) to 5 (“strongly agree”) was used to assess participants’ perceptions of anatomy learning with the AR app. Participants indicated their level of agreement or disagreement with the statements using this scale. The researchers conducted descriptive analysis to calculate the median and IQR as the data were derived from Likert-scale responses and were not normally distributed. An overall perception score was also computed by averaging individual item responses, from which the median and IQR were calculated. Then, inferential analysis was conducted using the Spearman rank-order correlation to explore the association between participants’ demographic variables and the overall perception score of the AR learning experiences. Spearman ρ was applied for all correlations since this test handles nonnormally distributed and ordinal data better, particularly in Likert scale-based studies. All the statistical tests were 2-sided, and a significance level of *P*<.05 was adopted.

## Results

### Participants’ Demographic

The results of the first section of the questionnaire, which gathered demographic information on the participants, are presented in Table 1. A total of 284 premedical students participated in the study, with 148 (52.1%) participants aged older than 18 years, 192 (67.6%) being female, and 60 (21%) participants having a monthly household income between MYR 1000 and MYR 3000 (US $251.95-US $755.86). Furthermore, 151 (53.2%) participants were from FiS Batch 29, who enrolled in the medical university college in August 2023.

**Table 1.** Participants’ demographic information (N=284).

Variable	Value, n (%)
Age (y)	
15‐16	2 (0.7)
17‐18	134 (47.2)
>18	148 (52.1)
Gender	
Male	92 (32.4)
Female	192 (67.6)
Student batch number	
FiS^a^ Batch 27	36 (12.7)
FiS Batch 28	97 (34.2)
FiS Batch 29	151 (53.2)
Monthly household income (MYR)	
<1000 (<US $251.95)	38 (13.4)
1000-3000 (US $251.95-US $755.86)	60 (21.1)
3001-5000 (US $755.87-US $1259.76)	42 (14.8)
5001-7000 (US $1259.77-US $1763.67)	53 (18.7)
7001-10,000 (US $1763.68-US $2519.53)	48 (16.9)
>10,000 (>US $2519.53)	43 (15.1)

a FiS: Foundation in Science.

### Evaluation Outcomes

An analysis of the results gathered from the second section of the questionnaire is presented in Table 2. The researchers analyzed the questionnaire items as a unified construct, and due to the ordinal nature and nonnormal distribution of the data, the participants’ responses were summarized using the median and IQR. For all items, the median score was 5.00 (IQR 4.00‐5.00), suggesting generally positive responses among respondents. For each participant, the composite mean of all Likert-scale items was used to calculate the overall perception score through the calculation of the median and IQR. The computed overall perception score reflected a high level of agreement, with a median of 4.92 (IQR 4.17‐5.00). These important findings suggest that the premedical students perceived the AR app as an interactive, engaging, and accessible supplementary tool for anatomy learning.

**Table 2.** Evaluation of outcomes of the Likert scale^a^.

Number	Items	Value, median (IQR)
1	I think the “anatomy learning app with AR^b^” is very interesting.	5.00 (4.00‐5.00)
2	I think “anatomy learning app with AR” is very helpful in learning human anatomy materials.	5.00 (4.00‐5.00)
3	In my opinion, “anatomy learning app with AR” is very usable in other fields.	5.00 (4.00‐5.00)
4	I think “anatomy learning app with AR” is helpful in visualizing the anatomy of the human body.	5.00 (4.25‐5.00)
5	I think “anatomy learning app with AR” is helpful in understanding more deeply about the anatomy of the human body.	5.00 (4.00‐5.00)
6	I think the “anatomy learning app with AR” is faster in understanding the anatomy of the human body.	5.00 (4.00‐5.00)
7	I think the function of “anatomy learning app with AR” to see the description of each organ is very helpful and interesting.	5.00 (4.00‐5.00)
8	I think the function of “anatomy learning app with AR” to rotate 3D model is very beneficial in anatomical observation.	5.00 (4.00‐5.00)
9	I think the function of “anatomy learning app with AR” to enlarge 3D model is very beneficial to see anatomy details.	5.00 (4.00‐5.00)
10	I think learning by “anatomy learning app with AR” is very practical in anatomy class.	5.00 (4.00‐5.00)
11	I am very interested in using “anatomy learning app with AR” anytime.	5.00 (4.00‐5.00)
12	Overall “anatomy learning app with AR” greatly aided the learning process.	5.00 (4.00‐5.00)

a Overall median perception score was 4.92 (IQR 4.17‐5.00).

b AR: augmented reality.

**Figure 7.**
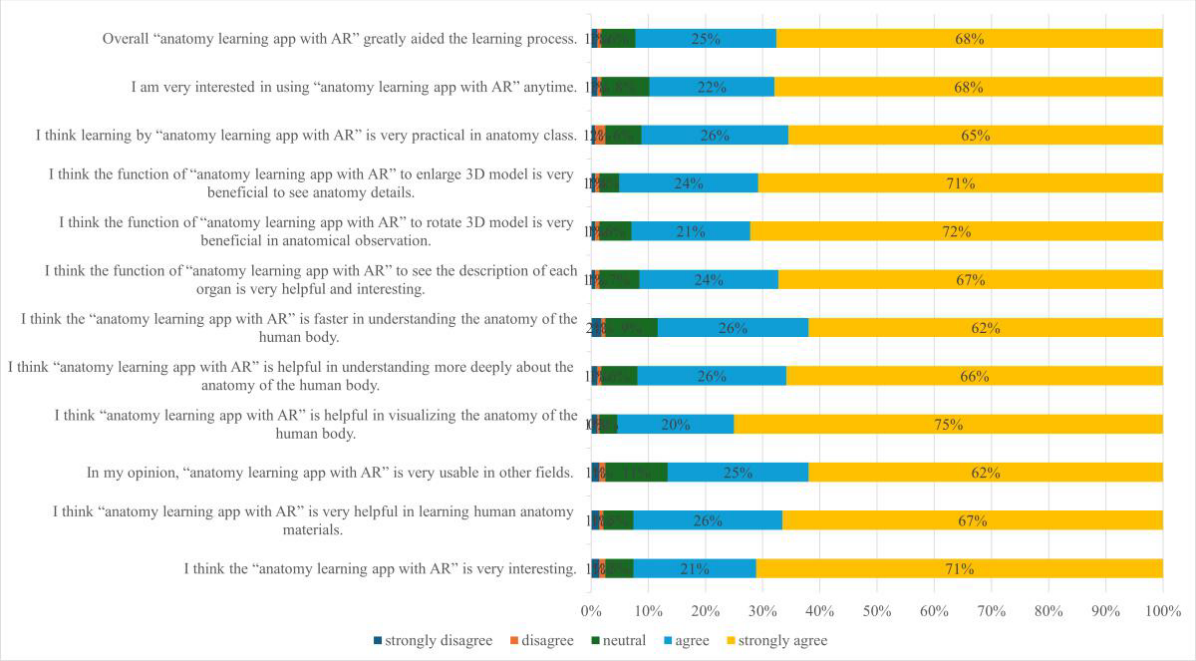
Participants’ perceptions of the anatomy learning app with augmented reality (AR). Different colors in the bars represent each distinct item on the Likert scale (1=“strongly disagree,” 2=“disagree,” 3=“neutral,” 4=“agree,” 5=“strongly agree”).

Figure 7 encapsulates the results from the 12 explicit statements which were responded to using the 5-point Likert scale. Overall, participants evaluated the anatomy learning experiences with AR positively. From a total of 284 participants, 202 (71%) strongly agreed, and 61 (21%) agreed that the anatomy learning app with AR is very interesting. Additionally, 213 (75%) participants strongly agreed, and 58 (20%) agreed that anatomy learning apps with AR are helpful in visualizing the anatomy of the human body. Furthermore, 205 (72%) participants strongly agreed, and 59 (21%) agreed that the function of the anatomy learning app with AR to rotate 3D models is very beneficial for anatomical observation. Finally, 201 (71%) participants strongly agreed, and 69 (24%) agreed that the function of the anatomy learning app with AR to enlarge 3D models is very beneficial for observing anatomical details.

Based on participants’ responses to the Likert scale, it was noted that both the median scores across individual questionnaire items and the overall perception score were at the upper end of the scale (item: median 5.00, IQR 4.00‐5.00; overall: median 4.92, IQR 4.17‐5.00), demonstrating that most participants selected option 4 (“agree”) and option 5 (“strongly agree”) in their responses.

Most participants agreed that the experiences of learning anatomy with AR were interesting and helpful in understanding anatomy materials and in visualizing the human body’s anatomy. Participants also rated items related to perceived usefulness and engagement favorably, indicating positive impressions of the AR app in anatomy learning. Besides, participants appreciated the ability to rotate the models for observation from multiple angles and the zooming function of the 3D models. Participants also agreed that the AR app’s practicality significantly assisted their overall learning process in the complex course of anatomy. These findings reflect participants’ immediate, self-reported perception feedback following a short-term exposure to the AR app. It is vital to highlight that these findings do not represent objective measures of learning outcomes, efficiency, or the long-term educational impact of the AR app in anatomy education.

### Correlations Between Demographic Variables and Overall Perception Scores

This section contains an analysis of the results obtained from the inferential statistics. Due to the ordinal nature of several demographic variables and the nonparametric distribution of the Likert scale data, the Spearman rank-order correlation was chosen for the inferential statistics. Table 3 presents a statistically significant correlation between gender and overall perception (ρ=0.212; *P*<.001), with female students reporting more favorable opinions of the AR app compared to male students. However, no statistically significant correlations were found between the overall perception score and age (ρ=0.034; *P*=.57), student batch number (ρ=−0.009; *P*=.88), or monthly household income (ρ=-0.041; *P*=.49), demonstrating that participants’ perceptions of AR-based learning were not influenced by their demographic variables.

These results indicated that while participants’ perception ratings of AR-based learning were mostly high across various demographic groups, female students had slightly more favorable responses compared to their male counterparts. Since no strong correlations existed with other demographic variables, this supports the comprehensive potential of AR-based learning in anatomy education.

**Table 3.** Correlation analysis (Spearman ρ and 2-tailed *P* value) between the overall perception scores and demographic information (N=284).

Variables	ρ	*P* value
Age (y)	0.034	.57
Gender	0.212^a^	<.001
Student batch number	−0.009	.88
Monthly household income (MYR)	−0.041	.49

a The correlation is significant at a significance level of .01 level (2-tailed).

## Discussion

### Principal Findings

Our findings demonstrate preliminary evidence that the integration of AR in early anatomy learning is positively perceived by premedical students. Following a structured 1-hour session, participants reported a favorable perception of the AR app as a potentially beneficial supplementary learning tool for anatomy education. These findings, however, are based on a single, short-term exposure and reflect students’ perceived value rather than objective measures of learning outcomes or sustained educational effectiveness. Demographic correlation analysis revealed that the overall perception score was not significantly associated with age, student batch number, or monthly household income, suggesting that AR’s positive reception was consistent across diverse students’ academic backgrounds and socioeconomic statuses. Although more than 50% of respondents were from a single batch (ie, FiS Batch 29), this distribution reflected student cohort size and participation rates, and no significant association was observed between student batch number and overall perception score, indicating that this imbalance did not substantially influence the findings. It also reflects that variations in the intake period did not influence students’ evaluation of the AR learning experience. However, it was noted that a statistically significant association existed between gender and perception, highlighting slight variations in how different gender subgroups interact with AR technology. Overall, these findings have answered the research question and supported the study hypothesis that premedical students perceive AR technology as a beneficial and acceptable pedagogical adjunct for anatomy learning, and this positive perception is universally shared and not strongly influenced by the premedical students’ demographic factors.

### Implications of Findings

From an educational viewpoint, this preliminary evidence supports the integration of AR as a supplementary tool rather than a substitution for traditional anatomy teaching methods such as lectures, textbook images, and static anatomical models. AR may be a particularly valuable approach in resource-constrained settings where access to human body donors or advanced laboratory facilities is limited, offering students an interactive means to explore the relationships between anatomical structures. Even though this study evaluated AR within structured learning sessions, the accessibility of AR via personal mobile devices suggests potential for self-directed and distance learning, enabling students to engage with interactive 3D anatomical models outside the classroom. However, optimal use may still benefit from facilitated and in-person integration, particularly for learners at an early stage of anatomy education. Collectively, these findings indicate that AR functions as a versatile and flexible digital tool that complements conventional teaching approaches while also offering opportunities to extend learning beyond traditional classroom settings.

### Comparison With Literature

This study’s results reinforce the potential of AR in anatomy education. The high perception ratings evident in this study align with earlier findings reporting favorable student opinions toward AR-based anatomy learning tools, particularly in terms of perceived usefulness, support for visualization of 3D structures, and increased student engagement [25-27]. However, like many preliminary assessments, these findings are based on brief exposure and student self-reported perceptions rather than objective measures of learning outcomes. In contrast to studies involving prolonged or curriculum-integrated use of AR, the present findings demonstrate initial impressions following a structured, short-term AR-based anatomy learning session.

AR can facilitate interactive, dynamic, and spatially immersive learning by transforming traditional educational approaches that have long relied on static 2D representations and conventional modeling techniques. AR’s interactive nature significantly enhances visual learning experiences by allowing students to observe intricate processes in detail [28]. AR provides clear depictions of complex anatomical structures by offering detailed visualizations in 3D and enhancing their layout and functions. This capability aligns with the need for comprehensive teaching and learning materials that offer in-depth anatomical descriptions [29]. Students expressed confidence in the effectiveness of learning through 3D models, including those involving AR, for grasping anatomical concepts [30]. The high perception ratings in this study reflect AR’s potential to make learning more appealing, thereby boosting students’ enthusiasm and motivation. As a result, students who engage with AR apps are more likely to attain improved knowledge retention and practical competency [31,32], which are critical for ensuring safe clinical practice.

One of the standout benefits of using AR as an educational tool is its multimedia features. Traditional anatomy educational methods frequently struggle to efficiently convey the intricate spatial relationships [33], often leaving students with difficulties understanding 3D inter-relationships between anatomical structures [30,34,35]. AR addresses this challenge by improving spatial reasoning, thus allowing students to visualize abstract scientific phenomena [36], that are otherwise challenging to replicate in conventional settings [37]. Historically, anatomy educators relied on verbal explanations and static illustrations from textbooks and atlases, expecting students to imagine these structures in real-time. AR allows students to scrutinize intricate structures such as nerves and blood vessels in isolation, which is a task challenging to achieve with traditional human donor body dissections, as these structures often lose their form when separated from supporting tissues [38].

The interactive capabilities of AR foster deeper engagement and enhance visual and spatial thinking [39]. The ability to display animated 3D models offers a significant advantage over traditional text and static illustrations. The interactive nature of AR supports real-time exploration and detailed examinations, which is particularly beneficial for premedical students who need to memorize complex anatomical information. AR enables learners to visualize and manipulate intricate concepts in real time by overlaying digital content onto the physical environment [40-43]. AR apps, designed with features like assistive touch, enable students to enlarge or rotate specific organs in the human body. AR also facilitates the acquisition of knowledge about numerous anatomical structures by allowing students to choose specific regions or structures of interest [44]. A study used a mobile AR app developed by the authors to enable users to dynamically explore various parts of the human body in 3D, enhancing their overall educational experience [45]. Likewise, a mobile app with AR designed to enhance learning of the musculoskeletal system received positive evaluations from users. The findings demonstrated that the anatomy system could efficiently improve retention of anatomical concepts and students’ engagement [46].

One of the key strengths of AR for anatomy education is its practicability. The ability to use personal devices for learning promotes self-paced and flexible learning, which is crucial in today’s digital learning environment [38]. The incorporation of virtual and real environments offers intuitive interaction mechanisms and unparalleled realism, which is largely supported by advancements in smart devices [47,48]. These advancements allow students to retrieve anatomical information anytime and anywhere. AR technology’s accessibility extends to various personal devices such as iPads, mobile phones, laptops, and smartwatches, enhancing its affordability and ease of use [49]. This accessibility and flexibility are particularly advantageous for understanding complex systems that involve multiple muscle groups, such as eye movement, which students can explore from a student’s desk or home [38]. The ability to control the app and engage in self-practice promotes active learning and self-assessment [50]. As AR decreases cognitive load and enhances cognitive, meta-cognitive, and affective outcomes, it is gaining popularity in higher educational institutions [51]. Furthermore, university students frequently express confidence in embracing AR as an alternative to traditional learning methods, largely due to their high digital literacy and familiarity with tablets and smartphones [52]. Integrating AR into an anatomy curriculum may provide students with an engaging, flexible tool that enhances their overall learning experiences, preparing them for future professional challenges in the field of medicine.

### Strengths and Limitations

This study has several strengths that contribute to the credibility and relevance of its findings. A major strength is the large sample size, which exceeded the minimum requirement and included students from multiple cohorts and diverse socioeconomic backgrounds. This allowed us to evaluate the perception of AR across a broad premedical student population. In addition, the questionnaire demonstrated excellent internal consistency and strong construct validity, supporting its suitability for capturing students’ overall perception of AR integration.

Several limitations should also be acknowledged. First, the study focused exclusively on students’ self-reported perceptions and did not assess objective learning outcomes, such as examination performance, knowledge retention, or skill acquisition. While a positive perception is an important indicator of educational acceptability, it does not necessarily equate to improved learning. Future studies should incorporate preintervention and postintervention assessments or comparative designs to better evaluate the educational effectiveness of AR.

Second, the cross-sectional design and single, brief exposure to the AR app limit conclusions about its long-term impact. Students’ positive responses may have been influenced by the novelty of the technology, and it remains unclear whether similar levels of engagement and perceived benefits would be sustained with repeated use over time. Longitudinal studies examining continued AR use across multiple anatomy topics or semesters would provide valuable insights into its durability as a learning tool.

Third, this study was conducted at a single institution using a commercially available AR app focused on basic anatomy content. Therefore, the findings may not be fully generalizable to other institutions, curricular structures, or learner groups, particularly students at more advanced stages of medical training. Differences in instructional design, technological infrastructure, and faculty facilitation may influence how AR is experienced and perceived.

Fourth, although the survey instrument demonstrated strong internal consistency, other forms of validity, such as predictive or discriminant validity, were not examined. The exclusive use of quantitative survey data precluded deeper exploration of students’ cognitive processes, challenges, or usability concerns during AR engagement. Incorporating qualitative methods, such as focus group discussions or interviews, could provide deeper insights into how AR supports or potentially hinders anatomy learning.

Finally, practical considerations related to implementation should be considered. Although AR apps are becoming increasingly accessible through personal devices, issues such as licensing costs, device compatibility, and classroom logistics may impact scalability and equitable access, particularly in larger cohorts or resource-limited settings.

Overall, this study provides early, learner-centered evidence that AR is positively received as a supplementary tool in premedical anatomy education. Future research incorporating objective learning outcomes, longer follow-up periods, and multi-institutional designs will be important to further define the educational value of AR and guide its sustainable integration into medical education curricula.

### Conclusions

This study provided preliminary evidence that AR is consistently perceived as an interactive and engaging supplementary tool to traditional approaches in anatomy learning by premedical students. Students expressed high levels of receptivity to AR across various demographics, regardless of their age, previous educational background, or individual socioeconomic status. These findings support the incorporation of AR as a comprehensive tool in early anatomy learning. Importantly, this work provides preliminary evidence and serves as a foundation for larger-scale, multi-institutional studies. The app used in this study offers multifaceted benefits, including the enhancement of visualization and the enrichment of interest in anatomy learning. Future research involving multiple institutions, various learner cohorts, and incorporating objective assessments is warranted to validate and extend these findings and to further evaluate AR’s pedagogical efficacy in anatomy education.

## Supplementary material

10.2196/64402 Checklist 1 CHERRIES checklist.

## Abbreviations

AR: augmented reality
CHERRIES: Checklist for Reporting Results of Internet E-Surveys
FiS: Foundation in Science
MUCM: Manipal University College Malaysia
