# Supplementary material for: Perspectives of Premedical Students at a Medical University College in Malaysia Regarding Augmented Reality Integration in Anatomy Education: Cross-Sectional Study
Source: JMIR Med Educ. 2026 Jun 8;12:e64402. doi: 10.2196/64402 (PMC13245848; doi:10.2196/64402)
Supplement: Checklist 1 [file mededu-v12-e64402-s001.pdf]

Supplementary File 1

Checklist for Reporting Results of Internet E-Surveys (CHERRIES)

Item Category	Checklist Item	Description	Section
Design	Describe survey design	Cross-sectional online survey conducted among pre-medical students.	Methods-Study Design
IRB (Institutional Review Board) approval and informed consent process	IRB approval	Approved by Research Ethics Committee, Manipal University College Malaysia (MUCM/Research Ethics Committee–035/2023).	Methods-Ethical considerations
	Informed consent	Information provided prior to survey access; participation was voluntary and consent implied by survey completion.	Methods – Ethical considerations, Online Survey Administration
	Data protection	No personal identifiers collected, anonymized data with restricted access.	Methods – Ethical considerations
Development and pre-testing	Development	Questionnaire adapted from a previous study and aligned with study objectives.	Methods – Data Collection
	Testing	Pilot study conducted to assess clarity and reliability.	Methods – Data Collection Tool
Recruitment process and description of the sample having access to the questionnaire	Open survey versus closed survey	Closed survey accessible only to eligible students.	Methods – Online Survey Administration
	Contact mode	Google Forms.	Methods – Data Collection
	Advertising the survey	Electronic distribution to eligible students.	Methods – Study Design, Online Survey Administration

Survey administration	Web/E-mail	Google Forms.	Methods-Data Collection, Online Survey Administration
	Context	Not applicable because Google Forms was used as a closed platform.	Methods-Data Collection
	Mandatory/Voluntary	Voluntary.	Methods – Data Collection, Online Survey Administration
	Incentives	No incentive.	Methods – Online Survey Administration
	Time/Date	September 6, 2023, to January 9, 2024.	Methods-Data Collection
	Randomization of items or questionnaires	Both questionnaire items and response options were randomized using the built-in shuffle functions in Google Forms.	Methods-Data Collection
	Adaptive questioning	No: Adaptive questioning was not applied. All participants were presented with the same set of questionnaire items in a fixed structure, regardless of their responses to preceding questions.	N/A
	Number of Items	Twelve questionnaire items.	Methods-Data Collection Tool
	Number of screens (pages)	The survey has 5 pages.	Methods-Data Collection
	Completeness check	Yes, data completeness was assessed both during and after survey submission. As a dynamic completeness check, each question required a	Methods-Data Collection

		response before the respondent could proceed to the next page.	
	Review step	Back button and response editing were enabled.	Methods-Data Collection
Response rates	Unique site visitor	Responses were restricted to a single entry per participant.	Methods-Online Survey Administration
	View rate	Not available due to Google Forms limitations.	N/A
	Participation rate	Calculated based on eligible students and submissions.	Methods-Data Analysis
	Completion rate	Identical to participation rate due to mandatory completion.	Methods-Data Analysis
Preventing multiple entries from the same individual	Cookies used	To prevent multiple submissions, responses were restricted to a single entry per participant.	Methods- Online Survey Administration
	IP check	IP address checking was not performed. However, duplicate responses were prevented by limiting the survey to one response per participant.	N/A
	Log file analysis	No log file analysis was conducted. Duplicate entries were prevented by limiting the survey to one response per participant.	N/A
	Registration	Participants were required to sign in, and the survey was restricted to one response per account. The survey was not displayed again after submission, preventing duplicate entries.	N/A

Analysis	Handling of incomplete questionnaires	Only fully completed questionnaires were included in the final analysis.	Method-Data Analysis
	Questionnaires submitted with an atypical timestamp	No minimum or maximum completion time threshold was defined, and no responses were excluded based on completion time.	N/A
	Statistical correction	Descriptive statistics and Spearman’s rank-order correlation.	Method-Data Analysis
